# Associations between body mass index and gastroesophageal cancer incidence and mortality: novel insights from a nationwide registry-based cohort study

**DOI:** 10.1093/dote/doaf018

**Published:** 2025-03-17

**Authors:** Usman Saeed, Tor Åge Myklebust, Trude Eid Robsahm, Dagfinn Aune, Bjørn Møller, Bjørn Steen Skålhegg, Sheraz Yaqub, Tom Mala

**Affiliations:** Department of Gastrointestinal and Pediatric Surgery, Oslo University Hospital, Oslo, Norway; Department of Registration, The Cancer Registry of Norway, Norwegian Institute of Public Health, Oslo, Norway; Department of Research and Innovation, Møre and Romsdal Hospital Trust, Ålesund, Norway; Department of Research, The Cancer Registry of Norway, Norwegian Institute of Public Health, Oslo, Norway; Department of Research, The Cancer Registry of Norway, Norwegian Institute of Public Health, Oslo, Norway; Department of Epidemiology and Biostatistics, School of Public Health, Imperial College London, London, UK; Department of Nutrition, Oslo New University College, Oslo, Norway; Department of Registration, The Cancer Registry of Norway, Norwegian Institute of Public Health, Oslo, Norway; Division for Molecular Nutrition, Institute of Basic Medical Sciences, University of Oslo, Oslo, Norway; Department of Gastrointestinal and Pediatric Surgery, Oslo University Hospital, Oslo, Norway; Institute of Clinical Medicine, University of Oslo, Oslo, Norway; Department of Gastrointestinal and Pediatric Surgery, Oslo University Hospital, Oslo, Norway; Institute of Clinical Medicine, University of Oslo, Oslo, Norway

**Keywords:** body mass index, obesity, gastroesophageal cancer, esophageal adenocarcinoma, gastric adenocarcinoma, cancer incidence, cancer mortality, early-onset cancer

## Abstract

The association between body mass index (BMI) and cancers of the esophagus and the stomach remains complex and requires further exploration. This study aimed to investigate this association, including early-onset (< 50 years) cancer and cancer related mortality. A nationwide registry-based cohort study was performed by linking data from multiple national registries in Norway. The cohort included 1,723,692 individuals, with 22,473 gastroesophageal cancer cases identified over 55,701,169 person-years of follow-up. In men, a 5 kg/m^2^ increase in BMI was associated with an increased risk of esophageal (HR 1.34, 95%CI 1.22–1.48) and cardia adenocarcinoma (HR 1.36, 95% CI, 1.22–1.50). This finding extended to individuals with high BMI in early life (16–29 years) for esophageal adenocarcinoma. The highest risk per 5 kg/m^2^ increase in BMI was observed for early-onset esophageal (HR 2.49, 95%CI 1.23–5.02) and cardia adenocarcinoma (HR 2.26, 95%CI 1.19–4.27). Among women, increased BMI was associated with a higher risk of both esophageal (HR 1.28, 95%CI 1.13–1.44) and gastric adenocarcinoma (HR 1.04, 95%CI 1.01–1.07). Women with elevated BMI in early life also demonstrated increased risk for these cancers. In both sexes, a 5 kg/m^2^ increase in BMI was inversely associated with squamous cell carcinoma of the esophagus. No association was observed between BMI and risk of cancer-related mortality. This study highlights an elevated risk of gastroesophageal adenocarcinomas with increasing BMI, with notable sex, age, and site-specific variations. The findings also point to a heightened risk of early-onset esophageal and cardia adenocarcinoma in men with high BMI.

## Graphical Abstract

**Graphical Abstract:**
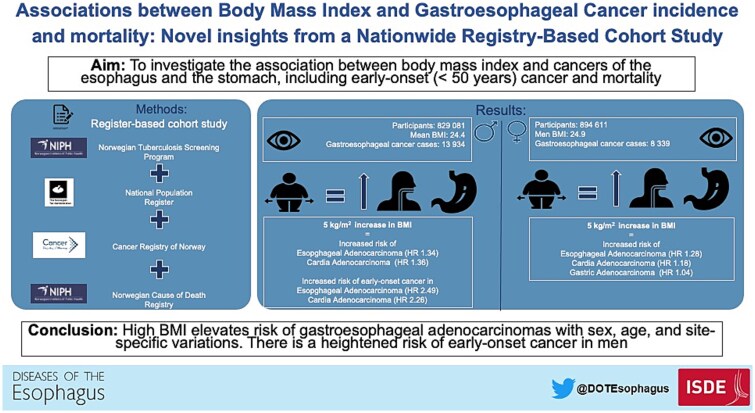

## INTRODUCTION

The association between high body mass index (BMI) and elevated cancer risk is robust for several malignancies.^1–5^ For esophageal and gastroesophageal junction adenocarcinoma several studies have shown a positive association with BMI. In contrast, esophageal squamous cell carcinoma (ESCC) exhibits an opposing trend.^6–10^ For gastric cancer, there is conflicting evidence if higher BMI increases cancer risk.^10–13^ Notably, there is a disparity in gastric subsite involvement, with a clearer association observed between BMI and cancer in the gastric cardia.^7^^,^^11^^,^^14–16^

The global incidence of esophageal cancer is on the rise, particularly in developing nations.^17^ This is partly attributed to the global rise in obesity, with approximately 18.1% of esophageal cancer deaths attributed to high BMI.^17^ Conversely, the incidence of gastric cancer has decreased in recent decades, primarily due to the eradication of *Helicobacter pylori* infections.^13^^,^^18^ Nonetheless, projections indicate sustained high incidence rates in certain regions and predict an increase in early-onset gastric cancer.^18^^,^^19^

For the association between cancer mortality and BMI, the literature is heterogeneous, with most studies focusing on peri-diagnostic BMI and its relationship with cancer-related survival and mortality, particularly in surgically treated patients.^20–24^ Findings either demonstrate no association on survival or suggest improved survival and reduced mortality risk with high peri-diagnostic BMI, a phenomenon termed the ‘obesity paradox in cancer’.^25^ However, the validity of these findings is questioned due to methodological limitations, including confounding factors, reverse causation from peri-diagnostic BMI measurement, and stratification biases.^16^^,^^25^^,^^26^

In this study, we aim to explore the association between BMI and the incidence and mortality of esophageal and gastric cancers, considering anatomical sites and histological subtypes, within a large Norwegian cohort with an extensive follow-up period. Secondary objectives include investigating the associations between these malignancies and BMI in young adulthood (16–29 years) and early-onset cancer (diagnosis at age < 50 years), and exploring population attributable fractions and changes in cancer incidence within the study period.

## METHODS

### Study design

This study is part of a research project exploring potential population-based associations between BMI, age, gender and cancer across a variety of cancer entities in Norway.^27^ A registry-based cohort study was conducted crosslinking several Norwegian registries based on the Norwegian Tuberculosis Screening Program (NTSP), a comprehensive nationwide unselected health survey conducted between 1963 and 1975. We extracted data on body height and weight measured by healthcare professionals and electronically recorded. These baseline data were linked to the Cancer Registry of Norway for data on cancer, the Population registry for data on vital status, and the Norwegian Cause of Death Registry for causes of death, leveraging the unique personal identification number assigned to all Norwegian residents. Detailed descriptions of the screening program and the registries have previously been published.^28^

### Study cohort and outcomes

Exclusions, outlined in Figure 2 and detailed in the Supplementary Methods S1, encompassed individuals with missing data, short stature,^29^ BMI less than 15 kg/m^2^ or greater than 50 kg/m^2^, age below 16 or above 75 years, lack of registered follow-up, and individuals diagnosed with any cancer within one year of NTSP screening.

Using the International Classification of Diseases (ICD), 10^th^ revision, and ICD for Oncology (ICD-O), 3rd edition, giving topography and morphology, cancers were categorized into esophageal adenocarcinoma (EAC), ESCC, esophagus and cardia adenocarcinoma (ECA), cardia adenocarcinoma (CA), gastric adenocarcinoma (GA), and non-cardia gastric adenocarcinoma (NCGA) (Supplementary Table S2 and S3). Data to define ECA, CA, and NCGA was only available in Cancer Registry of Norway after 1993, hence follow-up for these individuals with cancers classified according to the ICD-O-3 lexicon commenced in 1993.

To investigate the risk associated with high BMI during young adulthood, the cohort was stratified into age group 1, comprising individuals aged 16 to 29 years at the time of screening. To adjust for potential changes in BMI at the population level over time, three time periods based on the year of screening were delineated: 1963–1967, 1968–1971, and 1972–1975. No significant changes in BMI were observed across these time periods.^29^ Early-onset cancer was defined as cancer before the age of 50 years.^30^

### Statistical analyses

To present standard descriptive statistics, means and standard deviations were computed for continuous variables, while categorical variables were represented using absolute and relative frequencies. Analyses were conducted across both sexes combined, as well as separately for men and women.

For ECA we pooled esophagus and cardia adenocarcinoma for sensitivity analysis, as literature shows similar risk profile for both cancer subsite. Correspondingly, GA was categorized into CA and NCGA as current evidence indicate opposite risk profile regarding BMI.

### Incidence

Multivariable Cox proportional hazard regression models were estimated to investigate the association between BMI and cancer risk, adjusting for potential confounders such as age at screening and sex. BMI was treated as a continuous variable assuming log-linearity, enabling estimation of hazard ratios (HR) with 95% confidence intervals (CI) for every 5 kg/m^2^ increase in BMI.

To explore potential non-linear effects, BMI was modeled using restricted cubic splines with five degrees of freedom, allowing calculation of predicted HRs with 95% CI, illustrating smooth non-linear curves of HR as BMI increased.

Interaction terms between BMI and Age Group 1 were included to assess the impact of BMI during young adulthood. Analyses were also conducted to evaluate the risk of early-onset cancer with every 5 kg/m^2^ increase in BMI by censoring individuals at age 50.

Individuals were followed from the date of NTSP screening until any of the following events: diagnosis of the cancer of interest, reaching age 75, death, emigration, or administrative censoring on 31 December 2018. Attained age was utilized as the timescale in the analysis, given its significance as a key risk factor for cancer development.

### Cancer-related mortality

Multivariable Cox regression models were estimated to assess the effect of a 5 kg/m^2^ increase in pre-diagnostic BMI on cancer-specific mortality for all cancer subsites studied, adjusting for age at screening, age at cancer diagnosis, diagnostic time-period, and sex. Cancer stage adjustment was omitted considering its potential mediating effect. The outcome of interest was death caused by the cancer of interest. Individuals were followed from cancer diagnosis until death or administrative censoring, with time since diagnosis as the timescale.

### Other analysis

Population Attributable Fractions were computed for EAC and CA, categorizing BMI into underweight (<18.5), normal weight (18.5–24.9), and overweight (>25). Analysis was adjusted for age at screening. Current prevalence estimates for underweight and overweight were sourced from Statistics Norway.^31^

All statistical analyses were conducted utilizing Stata 18.0 software.^32^

### Ethics and approvals

This study was approved by the Regional Committee for Medical and Health Research Ethics in South-eastern Norway (Reference Number: 2018/670), with authorization from the data protection officer at Oslo University Hospital (Reference ID: SD0759843). Additional approvals were obtained from the Norwegian Institute of Public Health, Cancer Registry of Norway, and the Norwegian Tax Administration for National Population Register data access. Individual consent requirement was waived by the Regional Ethical Committee.

The STROBE guidelines for reporting of observational studies were followed.^33^

## RESULTS

**Fig. 1:**
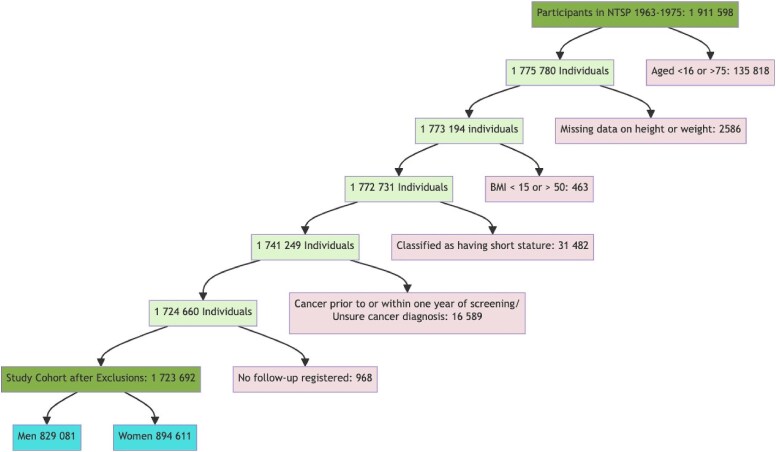
Flowchart of the study cohort from the Norwegian Tuberculosis Screening Program 1963–1975. ^*^BMI = Body Mass Index

The final study cohort consisted of 1,723,692 individuals, of whom 829,081 (48.1%) were men (Fig. 1). Baseline characteristics and patient demographics are provided in Table 1. During a follow-up period of 55,701,169 person-years, 22,473 cases of primary esophageal and gastric cancers were identified. Detailed characteristics of individuals diagnosed with cancer are outlined in Table 2.

**Table 1:** Characteristics of the study cohort retrieved from the Norwegian Tuberculosis Screening Program (1963–1975)

	**Men**	**Women**	**Both sexes**
**Study cohort**	829,081 (48.1%)	894,611 (51.9%)	1,723,692
**Age at measurement** ^†^ **(years), Mean (SD)**	43.2 (16.5)	43.1 (16.5)	43.2 (16.5)
**Height (cm), Mean (SD)**	175.2 (6.3)	162.5 (5.8)	168.6 (8.8)
**Weight (kg), Mean (SD)**	74.8 (10.6)	65.6 (11.4)	70.1 (11.9)
**BMI** ^‡^ **(kg/m**^**2**^**) Mean (SD)**	24.4 (3.2)	24.9 (4.4)	24.6 (3.9)
***BMI categories (kg/m*** ^***2***^ ***)*** ^***§***^			
Underweight (BMI 15–18.5)	12,297 (1.5%)	23,817 (2.7%)	36,114 (2.1%)
Normal (18.5–25)	494,357 (59.6%)	494,551 (55.2%)	988,908 (57.4%)
Overweight^25–30^	282,710 (34.1%)	262,828 (29.4%)	545,538 (31.6%)
Obese (>30)	39,717 (4.8%)	113,415 (12.7%)	153,132 (8.9%)
***Age groups at measurement*** ^†^ ***(years)***			
Age group 1 (16–29)	216,228 (26.1%)	234,892 (26.3%)	451,120 (26.1%)

Values are N (%) unless otherwise stated.

^†^Initial BMI measurement

^‡^Body Mass Index

^§^WHO classification.

**Table 2:** Characteristics of patients developing gastroesophageal cancers

	**Men**	**Women**	**Both sexes**
**Number of cancer cases**			
Esophagus adenocarcinoma^†^ Esophagus squamous cell carcinoma^†^ Esophagus and cardia adenocarcinoma^†^^,^^#^ Cardia adenocarcinoma^†^^,^^#^ Gastric Adenocarcinoma^†^ Non-cardia gastric adenocarcinoma^†^^,^^#^	1165 (76%) 1373 (66%) 2141 (74%) 1160 (72%) 11,396 (61%) 2041 (56%)	357 (24%) 711 (34%) 760 (26%) 455 (28%) 7271 (39%) 1620 (44%)	1522 2084 2901 1615 18,667 3661
**Years of follow up** ^‡^ **, Mean (SD)**			
Esophagus adenocarcinoma^†^ Esophagus squamous cell carcinoma^†^ Esophagus and cardia adenocarcinoma^†^^,^^#^ Cardia adenocarcinoma^†^^,^^#^ Gastric Adenocarcinoma^†^ Non-cardia gastric adenocarcinoma^†^^,^^#^	34.8 (10.4) 24.2 (12.9) 13.0 (7.1) 11.9 (7.2) 24.1 (12.8) 13.0 (7.1)	35.1 (10.2) 26.9 (12.8) 12.0 (6.9) 11.0 (6.8) 26.5 (12.6) 12.0 (6.9)	34.9 (10.4) 25.1 (12.9) 12.7 (7.1) 11.6 (7.1) 28.9 (12.8) 12.7 (7.1)
**Mean age at measurement (years)** ^§^, **Mean (SD)**			
Esophagus adenocarcinoma^†^ Esophagus squamous cell carcinoma^†^ Esophagus and cardia adenocarcinoma^†^^,^^#^ Cardia adenocarcinoma^†^^,^^#^ Gastric adenocarcinoma^†^ Non-cardia gastric adenocarcinoma^†^^,^^#^	37.7 (12.8) 45.0 (14.1) 35.8 (11.2) 37.7 (12.8) 45.0 (14.1) 35.8 (11.2)	41.2 (12.9) 46.1 (14.5) 39.6 (11.5) 42.0 (12.9) 46.1 (14.5) 39.6 (11.5)	38.7 (13.0) 45.4 (14.2) 36.8 (11.4) 38.7 (13.0) 45.4 (14.2) 36.8 (11.4)
**Mean age at diagnosis (years), Mean (SD)**			
Esophagus adenocarcinoma^†^ Esophagus squamous cell carcinoma^†^ Esophagus and cardia adenocarcinoma^†^^,^^#^ Cardia adenocarcinoma^†^^,^^#^ Gastric adenocarcinoma^†^ Non-cardia gastric adenocarcinoma^†^^,^^#^	71.9 (9.6) 68.9 (9.7) 72.0 (9.7) 72.0 (9.6) 68.7 (9.7) 72.0 (9.7)	76.6 (9.7) 72.5 (9.8) 75.5 (9.9) 76.6 (9.7) 72.5 (9.8) 75.5 (9.9)	73.1 (9.8) 70.0 (9.9) 72.9 (9.8) 73.1 (9.8) 70.0 (9.9) 73.9 (9.8)

All values are N (%) unless otherwise stated.

^†^Cancer classification is given in Supplementary Methods S1

^‡^Follow-up from BMI registration to cancer diagnoses.

^§^Initial BMI measurement

^#^Cancer diagnosis available from 1993—follow-up calculated from this date.

### Esophageal adenocarcinoma

**Fig. 2:**
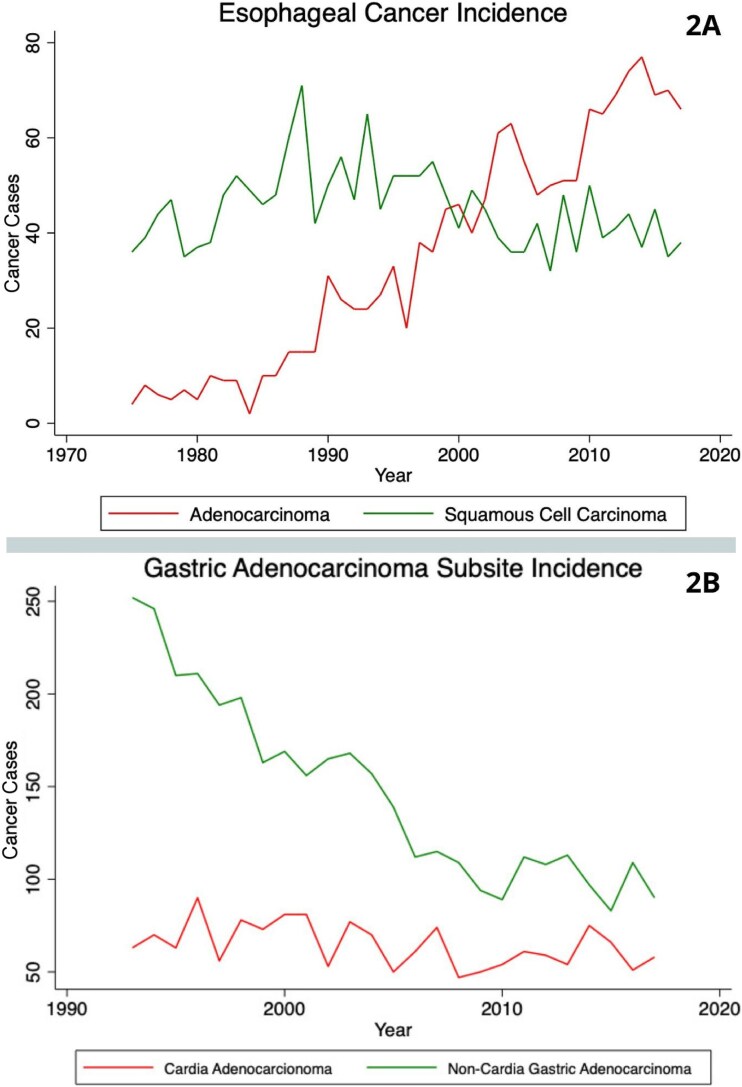
Trends in cancer incidence across the study period. (A) The incidence of esophageal adenocarcinomaand squamous cell carcinoma. (B) The incidence of gastric cardia adenocarcinoma and non-cardia gastric adenocarcinoma.

We observed 1522 EAC cases, of whom 357 were women (24%), over an average follow-up of 34.9 years (SD 10.4). The mean age at diagnosis was 73.1 years (SD 9.8) (Table 2). EAC incidence increased over the study period (Fig. 2A).

**Fig. 3:**
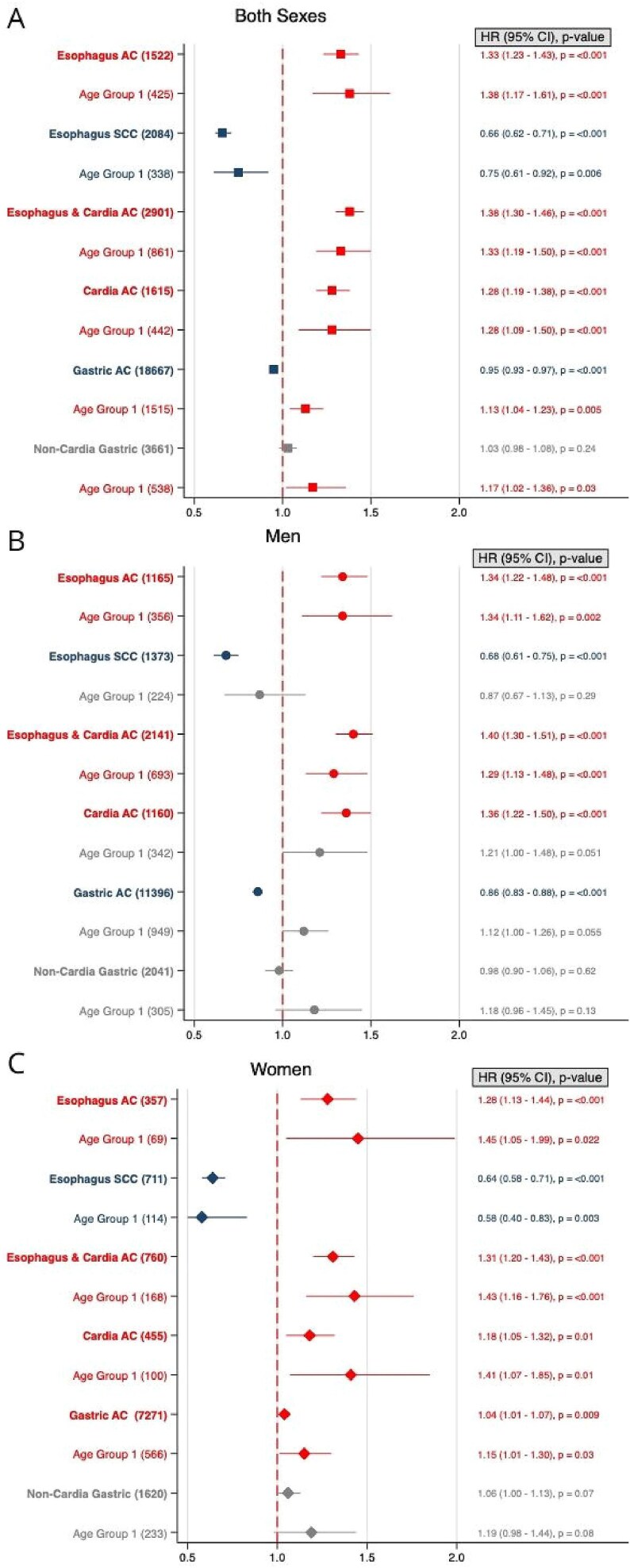
Forest plots showing hazard ratios (HR) with 95% confidence intervals (CI) for risk of gastroesophageal cancers by age groups for both sexes combined (A), men (B), and women (C). Five kg/m^2^ increase in body-mass index (BMI) is shown for each sex and BMI is fitted as a linear effect. Number of cancer cases are given in brackets. Age 16–29 was at time of BMI measurement. ▪ = Both sexes. • = Men. ♦ = Women.

**Fig. 4:**
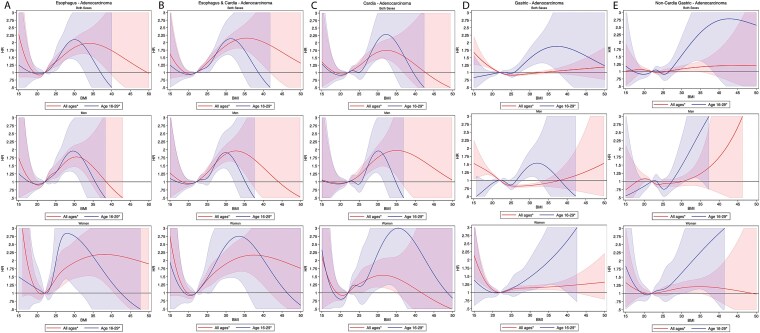
Age-adjusted hazard ratios (HR) for gastroesophageal cancers according to Body Mass Index (BMI). Curves are plotted for both sexes combined, men, and women for esophageal adenocarcinoma (A), pooled esophagus and cardia adenocarcinoma (B), gastric cardia adenocarcinoma (C), gastric adenocarcinoma (D), and non-cardia gastric adenocarcinoma (E) with 95% confidence intervals, allowing for non-linear effects modelled using restricted cubic splines. The reference BMI was fitted as a spline with HR fixed as 1·0 is 22 kg/m^2^.

For each 5 kg/m^2^ increase in BMI, HR for developing EAC was 1.33 (95% CI, 1.23–1.43) across the cohort and 1.38 (95% CI, 1.17–1.61) for individuals in Age Group 1. In men, the HR was 1.34 (95% CI, 1.22–1.48) for the overall cohort and 1.34 (95% CI, 1.11–1.62) in Age Group 1, while in women, it was 1.28 (95% CI, 1.13–1.44) for the overall cohort and 1.45 (95% CI, 1.05–1.99) in Age Group 1 (Fig. 3). Analysis using BMI as a non-linear covariate confirmed these trends (Fig. 4A).

**Fig. 5:**
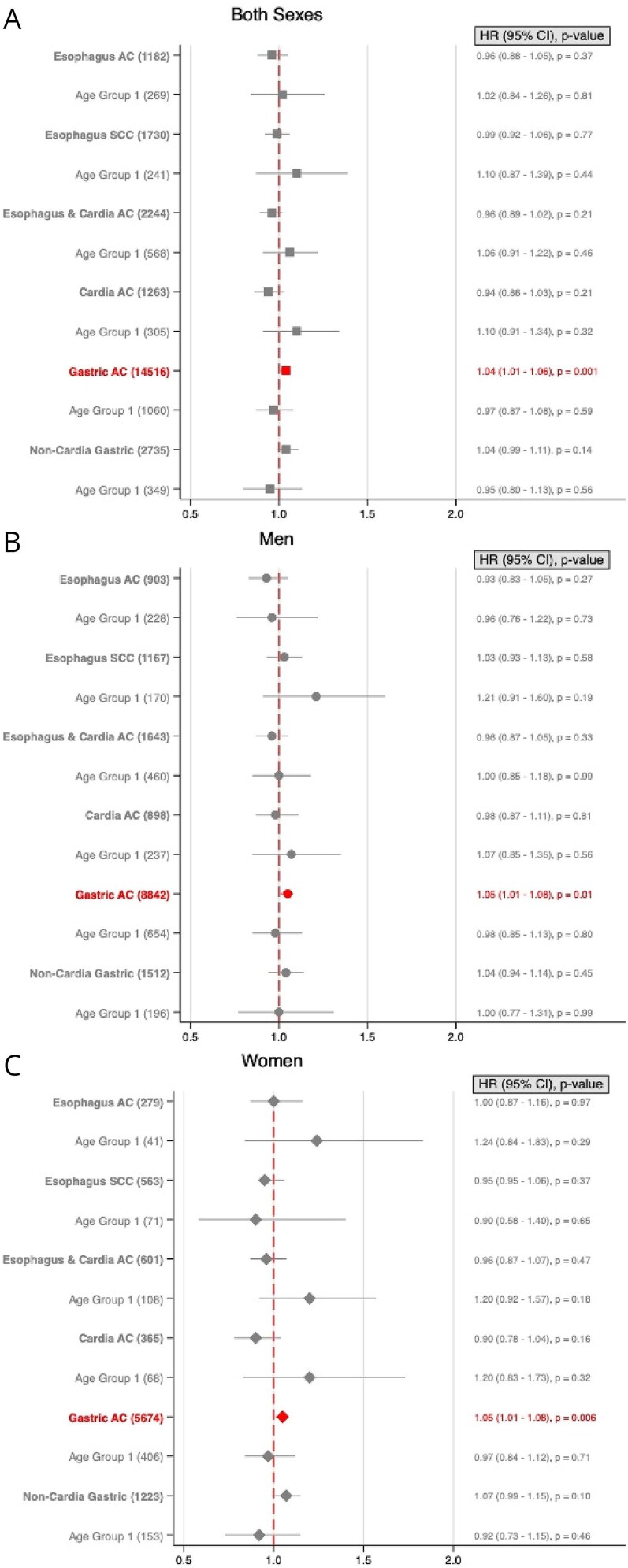
Forest plots showing hazard ratios (HR) with 95% confidence intervals (CI) for gastroesophageal cancer specific mortality by age groups for both sexes (A), men (B), and women (C). Five kg/m^2^ increase in body-mass index (BMI) is shown for each sex and BMI is fitted as a linear effect. Number of cases given in brackets. Age 16–29 was at time of BMI measurement. ▪ = Both sexes. • = Men. ♦ = Women.

**Fig. 6:**
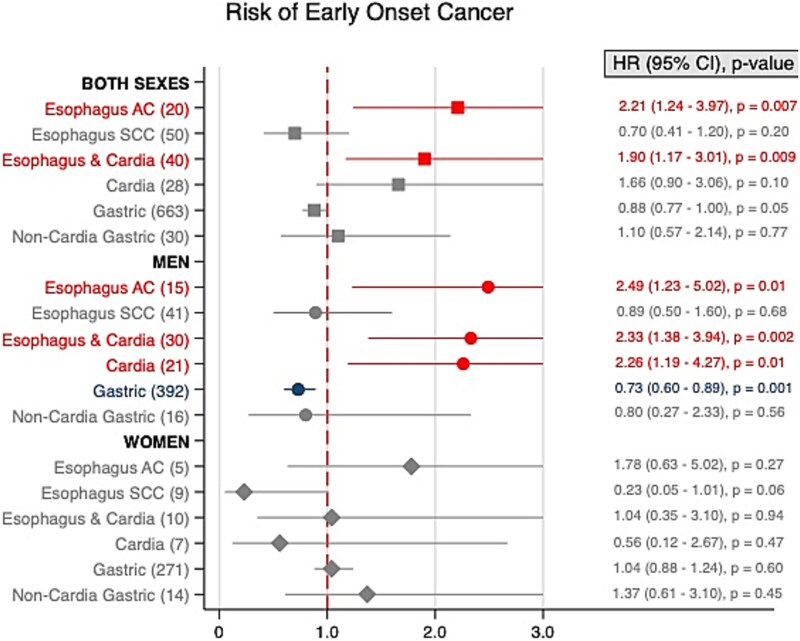
Forest plots showing hazard ratios (HR) with 95% confidence intervals (CI) for early-onset gastroesophageal cancer by age groups for both sexes (A), men (B), and women (C). Five kg/m^2^ increase in body-mass index (BMI) is shown for each sex and BMI is fitted as a linear effect. Number of cases given in brackets. ▪ = Both sexes. • = Men. ♦ = Women.

There was no increased risk of EAC-related mortality in either sex (Fig. 5). However, a 5 kg/m^2^ increase in BMI was associated with a higher risk of early-onset EAC with an HR of 2.21 (95% CI, 1.24–3.97) for the overall cohort, though this elevated risk was significant only in men (HR 2.49, 95% CI, 1.23–5.02) (Fig. 6). Population attributable fractions for EAC was estimated at 19.4% for men and 26.0% for women.

### Esophageal squamous cell carcinoma

A total of 2084 ESCC cases were identified, with a mean follow-up of 25.1 years (SD 12.9), and 711 cases (34%) occurring in women. The mean age at diagnosis was 70.0 years (SD 9.9) (Table 2). ESCC incidence remained stable throughout the study period (Fig. 2A).

Each 5 kg/m^2^ increase in BMI was associated with a decreased risk of ESCC in the entire cohort (HR 0.66, 95% CI, 0.62–0.71) and in age group 1 (HR 0.75, 95% CI, 0.61–0.92). For women, this association was significant in both the overall cohort (HR 0.64, 95% CI, 0.58–0.71) and Age Group 1 (HR 0.58, 95% CI, 0.40–0.83), while in men, the negative association was only significant in the overall male cohort (HR 0.68, 95% CI, 0.61–0.75) (Fig. 3).

BMI was not associated with increased risk of ESCC-related mortality (Fig. 5) nor with early-onset ESCC for either sex (Fig. 6).

### Esophagus and Cardia Adenocarcinoma

There were 2901 ECA cases, 760 (26%) in women, during a mean follow-up period of 12.7 years (SD 7.1). The mean age at diagnosis was 72.9 years (SD 9.8).

A 5 kg/m^2^ increase in BMI was associated with an increased ECA risk in the overall cohort (HR 1.38, 95% CI, 1.30–1.46) and in age group 1 (HR 1.33, 95% CI, 1.19–1.50). This association held true for both men (HR 1.40, 95% CI, 1.30–1.51; HR 1.29, 95% CI, 1.13–1.48) and women (HR 1.31, 95% CI, 1.20–1.43; HR 1.43, 95% CI, 1.16–1.76) (Fig. 3). Non-linear analysis of BMI confirmed these associations (Fig. 4B).

No significant increase in ECA-related mortality with BMI was observed (Fig. 5). A 5 kg/m^2^ increase in BMI was associated with an elevated risk of early-onset ECA in men (HR 2.33, 95% CI, 1.38–3.94), but not in women (Fig. 6).

### Cardia adenocarcinoma

We observed 1615 CA cases, including 455 (28%) in women, over an average follow-up of 11.6 years (SD 7.1). The mean age at diagnosis was 73.1 years (SD 9.8). CA incidence remained stable throughout the study period (Fig. 2B).

A 5 kg/m^2^ increase in BMI was associated with an elevated CA risk in the entire cohort (HR 1.28, 95% CI, 1.19–1.38) and in age group 1 (HR 1.29, 95% CI, 1.09–1.50). This association was significant in women across the whole cohort (HR 1.18, 95% CI, 1.05–1.32) and in age group 1 (HR 1.41, 95% CI, 1.07–1.85), while in men, a significant increase was only observed in the overall cohort (HR 1.36, 95% CI, 1.22–1.50) (Fig. 3). Non-linear BMI analysis confirmed these findings (Fig. 4C).

There was no increased risk of CA-related mortality with BMI (Fig. 5). Increased BMI was associated with a heightened early-onset CA risk in men (HR 2.26, 95% CI, 1.19–4.27) but not in women or the overall cohort (Fig. 6). Population Attributable Fractions for CA was estimated at 13.7% in men and 3.7% in women.

### Gastric adenocarcinoma

Among 18,667 GA cases, 7271 (39%) occurred in women, with an average follow-up of 28.9 years (SD 12.8). The mean age at diagnosis was 70.0 years (SD 9.9).

In the entire cohort, each 5 kg/m^2^ BMI increase was associated with a slight reduction in GA risk (HR 0.95, 95% CI, 0.93–0.97). In age group 1, however, the risk increased slightly (HR 1.13, 95% CI, 1.04–1.23) (Fig. 3). Sub-analyses by sex revealed a decreased risk in men for the whole cohort (HR 0.86, 95% CI, 0.86–0.88) but no significant association in Age Group 1. In women, there was a slight increase in risk across the cohort (HR 1.04, 95% CI, 1.01–1.07) and in Age Group 1 (HR 1.15, 95% CI, 1.01–1.30). Non-linear analysis confirmed these patterns (Fig. 4D).

An elevated risk of GA-related mortality was observed with each 5 kg/m^2^ increase in BMI (HR 1.04, 95%CI 1.01–1.06), which was true for both men (HR 1.05, 95%CI 1.01–1.08) and in women (HR 1.05, 95%CI 1.01–1.08) (Fig. 5). No significant early-onset GA risk was found for either sex (Fig. 6). Population Attributable Fractions for GA was estimated at −10.0% for men and − 1.44% for women.

### Non-cardia gastric adenocarcinoma

A total of 3661 NCGA cases were observed, with a mean follow-up of 12.7 years (SD 7.1), and 1620 cases (44%) in women. The mean age at diagnosis was 73.8 years (SD 9.8). NCGA incidence declined throughout the study period (Fig. 2B).

There was no significant association between NCGA, and 5 kg/m^2^ BMI increase in the entire cohort (Fig. 3). However, age group 1 showed an elevated risk (HR 1.17, 95% CI, 1.02–1.36). By sex, no significant correlation was found between BMI and NCGA risk in either men including or women, including age group 1. Non-linear analysis supported these findings (Fig. 4E). No increased risk of NCGA-related mortality was observed (Fig. 5), nor was early-onset NCGA risk significant for either sex (Fig. 6).

## DISCUSSION

This study with unprecedented high number of cancer cases, examined the association between BMI and risks for esophageal and gastric cancers, revealing significant risk variations across cancer subtype, sex, age at BMI measurement, BMI levels, and age at cancer onset. The findings underscore and expands on available evidence that higher BMI correlates with increased risks of EAC, and CA, particularly for early-onset cancer. Conversely, a higher BMI was associated with a reduced risk of ESCC and, to a lesser degree, GA, except in younger individuals who displayed a modest increased risk. GA subsite analysis revealed no association between high BMI and NCGA.

### BMI and esophageal cancer risk

The incidence of EAC has increased markedly over the past five decades, largely attributed to the rising prevalence of obesity.^34^ Our study reflects this rise in EAC incidence (Fig. 2A). The population attributable fractions for obesity-associated EAC indicates that 19.4% of EAC cases in men and 26.0% in women could be attributed to high BMI. The prevalence of obesity in our cohort was 8.9% at time of the NTSP, a figure that has now increased to an estimated 23% in Norway.^35^ The current obesity-related population attributable fractions for EAC is thus likely to be higher.

Our study identified a clear positive association between increasing BMI and EAC risk across both sexes and in younger individuals consistent with prior research (Figs 3 and 4).^2^^,^^4–8^ Notably, both the International Agency for Research on Cancer (IARC) and the World Cancer Research Fund (WCRF) list EAC as an obesity-associated cancer.^1^^,^^3^ BMI mediated effects on EAC pathogenesis may be related to gastroesophageal reflux disease (GERD) and systemic inflammation.^36^ GERD, associated with high BMI, contributes to development of Barrett’s esophagus, a precursor of EAC.^36^ Interestingly, and substantiating the link between BMI and esophageal cancer, recent research has shown a reduced EAC risk after bariatric surgery inducing significant and sustained weight loss.^37^

In contrast, the inverse association between BMI and ESCC (Fig. 3) suggests that obesity does not contribute to ESCC and may even offer some protective effect, in line with previous studies.^8^ ESCC is less influenced by GERD but strongly associated with smoking and alcohol consumption, which are inversely correlated with BMI.^8^ Unfortunately, our data did not include information on smoking or alcohol use, preventing us to control for these potential confounders.

### BMI and gastric cancer risk

EAC and GA may share a common origin from similar gastric stem cell populations, following a shared oncogenic pathway driven primarily by chronic inflammation.^38^ The difference lies primarily in the etiology of the inflammation, where EAC and CA are linked to inflammation resulting from acid reflux and GERD, both of which have strong associations with high BMI. In contrast, NCGA is primarily linked to chronic inflammation and *H. pylori* infection.^38^ The eradication of *H. pylori* and increased incidence of GERD may contribute to the observed reduction in NCGA incidence and the concurrent rise in EAC incidence (Fig. 2). This distinction is reflected in our estimated population attributable fractions for obesity in these cancer subtypes. While the population attributable fraction for obesity-related EAC and CA in men was 19.4% and 13.7% respectively, the equivalent for GA was estimated at −10%.

Both the IARC and WCRF list gastric cardia cancer as associated with obesity,^1^^,^^3^ and a recent large umbrella review found strong evidence linking adiposity specifically to CA rather than to NGCA.^4^ A large Swedish cohort study also reported a positive association between BMI and gastric cardia cancer, with no significant association observed for NGCA.^5^ Our findings align with these reports (Fig. 3).

Our results suggest a reduced risk of GA for men in the whole cohort with increasing BMI (HR 0.86), consistent with the Swedish pooled cohort.^5^ Conversely, younger individuals (ages 16–29) showed a modestly increased GA risk (HR 1.13), particularly among women (HR 1.15). This trend corresponds with findings from a separate Swedish study, which demonstrated an 8% increased risk in a linear analysis, and a large cohort study based on the PLCO Cancer Screening cohort in the United States.^10^^,^^39^ This risk is predominately driven by a substantially increased risk for CA (HR 1.36). Even though no significant increased risk for GA was found in women, there was an increased risk for CA (HR 1.18). Furthermore, for CA a higher BMI-associated risk was observed in younger women, but not in younger men. This novel age-related finding (Figs 3 and 4) may reflect BMI-associated risks that evolve over the lifespan, or differences across generations in environmental or lifestyle factors impacting risk at different ages.

### Cancer related mortality

The literature on gastroesophageal cancer mortality and BMI is diverse, with most studies focusing on BMI at time of diagnosis, particularly among surgically treated patients.^20–22^ To our knowledge, no prior studies have specifically examined the association between pre-diagnostic BMI and cancer-related mortality, that may better reflect BMI as a risk factor.

Our findings suggest no significant or clinically relevant association between pre-diagnostic BMI and cancer-related death for any of the cancers examined (Fig. 5). The risk increase in gastric cancer mortality is minimal. This absence of association could represent a novel insight into the role of BMI on gastroesophageal cancer outcome.

### Early-onset cancer

The rise of early-onset (diagnosis <50 years) cancer has been notable with data from the Global Burden of Disease database highlighting a 79.1% increase in early-onset cancer incidence between 1990 and 2019.^40^ Specifically, early-onset EAC has increased by 2.9% annually in the U.S. and typically presents at a more advanced stage of disease.^41^ A similar trend is observed in early-onset GA where incidence has surged in recent decades, with early-onset GA now comprising over 30% of all gastric cancer cases in the United States.^42^

Research on the relationship between BMI and early-onset gastroesophageal cancer remains limited. A cross-sectional study from California reported a trend toward younger ages at diagnosis for both esophageal and gastric cancer, though they did not specifically examine cancers diagnosed before age 50.^43^ A pooled analysis of case–control studies reported a stronger association between both obesity and gastroesophageal reflux disease and early-onset esophageal adenocarcinoma than for diagnosis at later ages.^44^ In this regard, our study provides a novel contribution by identifying a clear association between elevated BMI and an increased risk of early-onset gastroesophageal cancers, particularly EAC and CA in men (Fig. 6). Our findings indicate that men with higher BMI showcased an almost 2.5-fold increased risk for both early-onset EAC and early-onset CA, suggesting that the rising incidence of early-onset cancers could, in part, be driven by the obesity epidemic.

### Strengths and limitations

Strengths of this study include the use of data from high-quality national registries, the large cohort size and the long follow-up enabling robust statistical power, and the ability to assess long-term risk trends cross various cancer subtypes. Additionally, our stratified analyses by age and sex allow for a nuanced understanding of BMI-associated cancer risks relevant for targeted preventive measures. Analyses were restricted to adenocarcinomas and squamous cell carcinomas only to reduce tumor heterogeneity. There was no major selection bias in the NTSP, and the chance of reverse causality is eliminated with BMI measurements years before cancer diagnosis. Finally, height and weight were measured and registered by health personal using standardized tools, minimizing the risk of recall bias and social desirability bias.

Limitations include BMI measurement at baseline only. Some individuals may have experienced significant weight fluctuations or adopted lifestyles that could alter their BMI-related risk, particularly for age-related analyses. However, several studies have shown increase in weight and BMI at a population level by increasing age and over time.^45^ In a small population of our cohort two BMI measurement were available, which showed an increase in BMI over time.^28^ BMI does not reflect body composition or fat distribution, which may play an independent role in cancer risk. Classification of proximal gastric, distal esophagus and junctional cancer has varied over time and are associated with some uncertainty. *Furthermore, we acknowledge the absence of data on major potential confounding factors, such as H. pylori* status, dietary habits, evidence of GERD, and smoking, which may influence the observed associations.

Lastly, the study cohort consists of a homogenous Norwegian population at the time of NTSP. This may limit the generalizability of findings to other populations with differing lifestyle or genetic risk profiles. Previous studies reveal stronger associations with BMI in Western countries compared to Asian.^13^^,^^16^

## CONCLUSION

Our study expands upon available knowledge and provides novel insights into the complex relationship between BMI and the risk of gastroesophageal cancers. A higher BMI was positively associated with increased risks of esophageal and cardia adenocarcinoma, particularly in early-onset cancer, while showing minimal to no association with non-cardia gastric adenocarcinoma. Additionally, our findings indicate no significant link between pre-diagnostic BMI and cancer-related mortality, providing new insights into the obesity-cancer outcome relationship.

## Supplementary Material

Supplementary_Material_doaf018

## Data Availability

The dataset used for this study is not publicly available due to the conditions set out in the General Data Protection Regulation (GDPR) and Norwegian law. Data sharing provides legal basis and conditions set out in applicable law. Data from the Norwegian heath registries apply via Helsedata.no.

## Notes

Reporting checklist: The authors have completed the STROBE reporting checklist.

Funding: No funding is received for this study.

Ethical statement: This study is approved by the Regional Ethical Committee for Medical and Health Research in South-eastern Norway (REC#: 2018/670) and the Oslo University Hospital data protection officer (SD0759843), alongside the Norwegian Institute of Public Health (NTSP data), the CRN and the Norwegian Tax Administration (the National Population Register data). Consent for using register data was waived by the Regional Ethical Committee for Medical and Health Research.

Specific author contributions: Usman Saeed (Conceptualization, Data curation, Formal analysis, Investigation, Methodology, Project administration, Validation), Tor Åge Myklebust (Conceptualization, Data curation, Formal analysis, Methodology), Trude Eid Robsahm (Conceptualization), Bjørn Møller (Conceptualization), Bjørn Steen Skàlhegg (Conceptualization, Supervision), Sheraz Yaqub (Conceptualization, Project administration, Supervision, Validation), Tom Mala (Conceptualization, Project administration, Supervision).
