# Supplementary material for: Associations between body mass index and gastroesophageal cancer incidence and mortality: novel insights from a nationwide registry-based cohort study
Source: Dis Esophagus. 2025 Mar 17;38(2):doaf018. doi: 10.1093/dote/doaf018 (PMC11911121; doi:10.1093/dote/doaf018)
Supplement: Supplementary_Material_doaf018 [file supplementary_material_doaf018.docx]

# Supplementary Methods S1

Study population and outcomes

Exclusions

- Cancer registry of Norway includes data on premalignant conditions and data on instances where the cancer diagnoses is unsure, hence patients with those instances were excluded
- Persons with short stature were excluded as height component of BMI is drastically reduced compared to weight in persons with short stature, hence the BMI values become not valid.
- Individuals diagnosed with cancer within one year of registration in NTSP were not included, as this could reflect a pre-existing disease affecting the weight (reverse causality)

# Supplementary Table S2

| Adenocarcinoma | | Squamous Cell Carcinoma |
| --- | --- | --- |
| 8140 | 8260 | 8070 |
| 8141 | 8261 | 8071 |
| 8142 | 8262 | 8072 |
| 8143 | 8263 | 8073 |
| 8144 | 8310 | 8074 |
| 8145 | 8450 | 8051 |
| 8148 | 8470 | 8123 |
| 8160 | 8471 | 8430 |
| 9161 | 7472 | 8560 |
| 8162 | 8480 | 8570 |
| 8251 | 8481 |  |
| 8574 | 8490 |  |
| 8200 | 8500 |  |
| 8210 | 8510 |  |
| 8211 | 8512 |  |
| 8213 | 8440 |  |
| 8221 | 8550 |  |
| 8231 | 8572 |  |
| 8244 | 8574 |  |
| 8255 | 8460 |  |

**Supplementary Table S2:** ICD-O-3 morphology codes grouping to define Adenocarcinomas and Squamous Cell Carcinomas

# Supplementary Table S3

| Cancer site | ICD-10 | ICD-O-3 Topography | Histology* |
| --- | --- | --- | --- |
| Esophagus Adenocarcinoma | C15 | - | Adenocarcinoma |
| Esophagus Squamous Cell Carcinoma | C15 | - | Squamous Cell Carcinoma |
| Esophagus & Cardia | - | 153+154+155+158+159+160 | Adenocarcinoma |
| Cardia | - | 160 | Adenocarcinoma |
| Stomach | C16 | - | Adenocarcinoma |
| Stomach w/o Cardia | - | 161+162+163+164+165+166 | Adenocarcinoma |

**Supplementary Table S3:** Cancer definitions using ICD10 and ICD-0-3 data available in the Cancer Registry of Norway

*Grouping of Adenocarcinoma and Squamous Cell Carcinoma utilizing ICD-0-3 morphology codes is given in supplementary table S1
